# Millimeter-scale soft capsules for sampling liquids in fluid-filled confined spaces

**DOI:** 10.1126/sciadv.adp2758

**Published:** 2024-08-28

**Authors:** Xiaoguang Dong, Boyang Xiao, Hieu Vu, Honglu Lin, Metin Sitti

**Affiliations:** ^1^ Department of Mechanical Engineering, Vanderbilt University, Nashville, TN 37235, USA.; ^2^ Physical Intelligence Department, Max Planck Institute for Intelligent Systems, 70569 Stuttgart, Germany.; ^3^ School of Medicine and College of Engineering, Koç University, 34450 Istanbul, Turkey.

## Abstract

Sampling liquids in small and confined spaces to retrieve chemicals and microbiomes could enable minimally invasive monitoring human physiological conditions for understanding disease development and allowing early screening. However, existing tools are either invasive or too large for sampling liquids in tortuous and narrow spaces. Here we report a fundamental liquid sampling mechanism that enables millimeter-scale soft capsules for sampling liquids in confined spaces. The miniature capsule is enabled by flexible magnetic valves and superabsorbent polymer, fully wirelessly controlled for on-demand fluid sampling. A group of miniature capsules could navigate in fluid-filled and confined spaces safely using a rolling locomotion. The integration of on-demand triggering, sampling, and sealing mechanism and the agile group locomotion allows us to demonstrate precise control of the soft capsules, navigating and sampling body fluids in a phantom and animal organ ex vivo, guided by ultrasound and x-ray medical imaging. The proposed mechanism and wirelessly controlled devices spur the next-generation technologies for minimally invasive disease diagnosis.

## Teaser

Millimeter-scale capsules enable sampling liquids in fluid-filled confined spaces for targeted liquid biopsy in disease diagnosis.

## INTRODUCTION

Body liquids—such as blood, bile, pancreatic juice, saliva, urine, mucus, and other liquids (*1*–*3*)—are rich resources of information regarding chemical composition, biomarkers, bacteria colonies, and other crucial components (*4*, *5*). This wealth of information helps researchers understand the mechanism of various diseases, such as cancer, and track the health condition of patients (*2*, *6*). For instance, targeted and local sampling of bile and pancreatic juice within the human body and their safe retrieval for external analysis can play a crucial role in early disease diagnosis (*7*). Over the past decade, bile and pancreatic juices have emerged as a promising source of potential biomarkers that can detect liver and pancreatic cancers in asymptomatic patients, with a wealth of cancer-specific proteins (*8*). For example, carbohydrate antigen 19-9 (CA 19-9) is considered the most extensively studied biomarker to date as a biomarker for tumor in pancreatic cancer (*9*). In addition, the body fluids also contain microbiota associated with diseases development (*10*), such as inflammatory bowel disease (*11*).

To collect these liquids, swallowable wireless capsule endoscope devices designed for the gastrointestinal (GI) tract (*12*–*14*) have been used for microbiota sampling in the GI tract. However, their overall sizes are on the centimeter scale (*15*–*19*), restricting their ability to access confined spaces at millimeter or smaller scales. This limitation hinders their ability to reach to intricate and tortuous structures, such as the bile and pancreatic ducts. In addition, flexible catheters, such as endoscopic retrograde cholangiopancreatography (ERCP) (*20*), could carry a miniaturized tube to access more confined spaces, such as the bile or pancreatic duct. However, the ERCP is invasive and has a high risk of complications including post-ERCP pancreatitis. In addition, ERCP also pose a substantial risk of contamination and causing gastric and duodenal secretion trauma and other complications (*21*–*23*). Therefore, it remains a challenge to access the tortuous lumen structures for collecting biofluids.

On the other hand, wireless mobile robots at the millimeter scale—propelled by external stimuli such as magnetic fields (*24*–*28*), acoustic fields (*29*, *30*), and other stimuli (*31*, *32*)—have demonstrated the capability to navigate in confined spaces owing to their diminutive size and agile locomotion. The use of untethered miniature robots has exhibited promise in the delivery of various chemicals and bioagents, such as drug, gene, and stem cells (*33*). However, the function of sampling liquids is now missing in millimeter-scale devices due to the absence of effective triggering and sealing mechanisms at small scales. For example, prior studies have showcased the effectiveness of submillimeter-sized magnetic grippers in conducting tissue biopsies in the porcine bile duct (*34*). However, the samples have a high risk of being contaminated during the locomotion as the samples remain unprotected or uncontained (*12*).

To tackle these current challenges, we report millimeter-scale soft capsules featuring hydrogel-and-elastomer hybrids controlled by external magnetic fields. These soft devices could be delivered and retrieved by a thin catheter and adeptly locomote in tubular structures inaccessible to the catheters. Their collective movement in a group not only amplifies sampling capacity but also enhances medical tracking performance and fault tolerance. The soft capsules have a specifically coated wetting property, allowing them to pump liquids inside efficiently. Our study systematically quantifies the triggering, pumping, and sealing performance of these soft capsules across a range of body fluids. Furthermore, we showcase the coordinated control of a group of soft capsules, navigating in a bile duct phantom and retrieving liquids. We also showcase sampling fluids in porcine liver ex vivo under the guidance of ultrasound and x-ray imaging. These elastomer-hydrogel soft capsules pave the way toward early detection of diseases within the human body.

## RESULTS

### Working principle of millimeter-scale soft capsules for liquid sampling

**Fig. 1.**
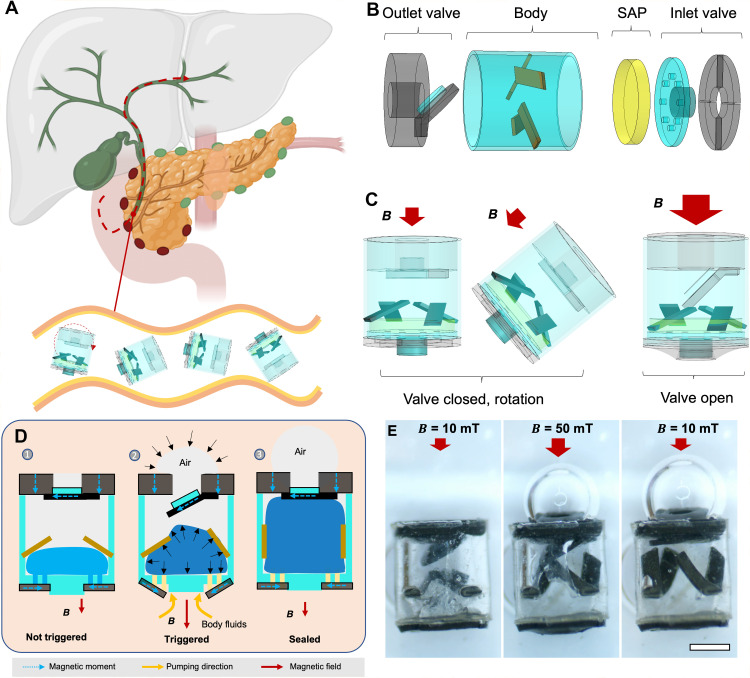
Wirelessly controlled millimeter-scale soft capsules for local liquid sampling in deep, tight and tortuous body sites. (**A**) Schematic of the capsule locomotion in these body sites. A group of magnetic soft capsules demonstrates controlled rolling on the surface within tubular structures without clustering. Created with BioRender. (**B**) Illustration of the components of the millimeter-scale soft capsule, featuring a hollow cylindrical body as a chamber and two magnetic valves for triggering and sealing. (**C**) Illustration of the decoupling between the capsule motion and valve opening by controlling magnetic field strength. (**D**) Illustration of the liquid sampling mechanism of the proposed soft capsule. The sampling process is triggered by an external magnetic field ***B***, and the sampling function is realized by the swelling effect of the SAP. (**E**) Optical images of the same soft capsule before sampling liquid and after completing the sampling process. To trigger the sampling, a magnetic field of about 70 mT, which is much higher than the locomotion magnetic fields, is applied to open the inlet valve. Scale bar, 500 μm.

As shown in Fig. 1A schematic, a collective of soft capsules exhibits controlled rolling motion on a substrate surface by applying a rotating external magnetic field. Despite the magnetic interaction leading to the formation of a chain among neighboring capsules, their separation is facilitated by the confinement of the boundary wall. When organized into a group, millimeter-scale soft capsules obtain the ability to navigate through fluid-filled and confined tubular structures inside organs, such as the bile duct, allowing further operation of sampling body fluids. Compared with existing tools, the primary advantage of using millimeter-scale capsules for liquid sampling is their safe and easy access to confined and tortuous structures, which is challenging and hazardous for conventional tools such as capsule endoscopes or flexible catheters to access.

Figure 1B illustrates the key components of a soft capsule for sampling water-based body liquids. The soft capsule has a hollow cylindrical structure made of polydimethylsiloxane (PDMS) as the capsule housing for liquid sampling, a super-absorbent polymer (SAP) (*35*, *36*) as a pump for liquid transportation, and two flexible magnetic valves for precise control over the triggering and sealing behaviors. First, to sample water-based fluids, sodium polyacrylate is used as the SAP (*35*, *37*), which is a cross-linked (network) polymer that contains sodium atoms and absorbs water by a process called osmosis. When in contact with water-based fluids, the sodium-containing polymer strives to maintain an equilibrium distribution of sodium between the network and water. The water-induced swelling of the polymer network occurs to balance sodium concentration, and the interlinked chains prevent dissolution or breaking apart in water. In addition, the two flexible magnetic valves are fully wirelessly controlled by external magnetic fields to regulate their opening and closing, thereby controlling the sampling and sealing functionalities. Figure 1C shows the decoupling between the valve opening and closing and the capsule rolling motion. When magnetic field is less than a threshold value, the valve remains closed while a rotating magnetic field steers the capsule to roll on a substrate as the net magnetic moment of the capsule aligns with the external magnetic field.

Figure 1D shows the operational principle of the soft capsule designed for on-demand liquid sampling through remote control of external magnetic fields. Initially, SAP is inserted inside the capsule body serving as the pump upon contact with water-based liquids (*38*). This action expels the air bubbles from the capsule body. Subsequently, two magnetic valves composed of magnetic-elastic composite materials referred to as inlet and outlet valves are precisely controlled by external magnetic fields. The deformation of these valves is controlled through external magnetic fields, allowing the triggering of liquid sampling and the sealing of the capsule to prevent contamination. The sampling process is triggered when the external magnetic field surpasses a threshold value *B*_0_, causing the inlet valve to open. Conversely, the sampling process is halted by reducing the magnetic field below *B*_0_. In Fig. 1E, an illustrative soft capsule with dimensions of 1.8 mm in height and 1.6 mm in diameter (see fig. S1 for the marked dimension of the capsule). The soft capsule demonstrated sampling deionized (DI) water as an example, wherein the swollen SAP pushing the soft valve to close, ensuring the safe preservation of the liquid sample within the polymer matrix by reducing the magnetic field strength.

### Design and fabrication of soft capsules

**Fig. 2.**
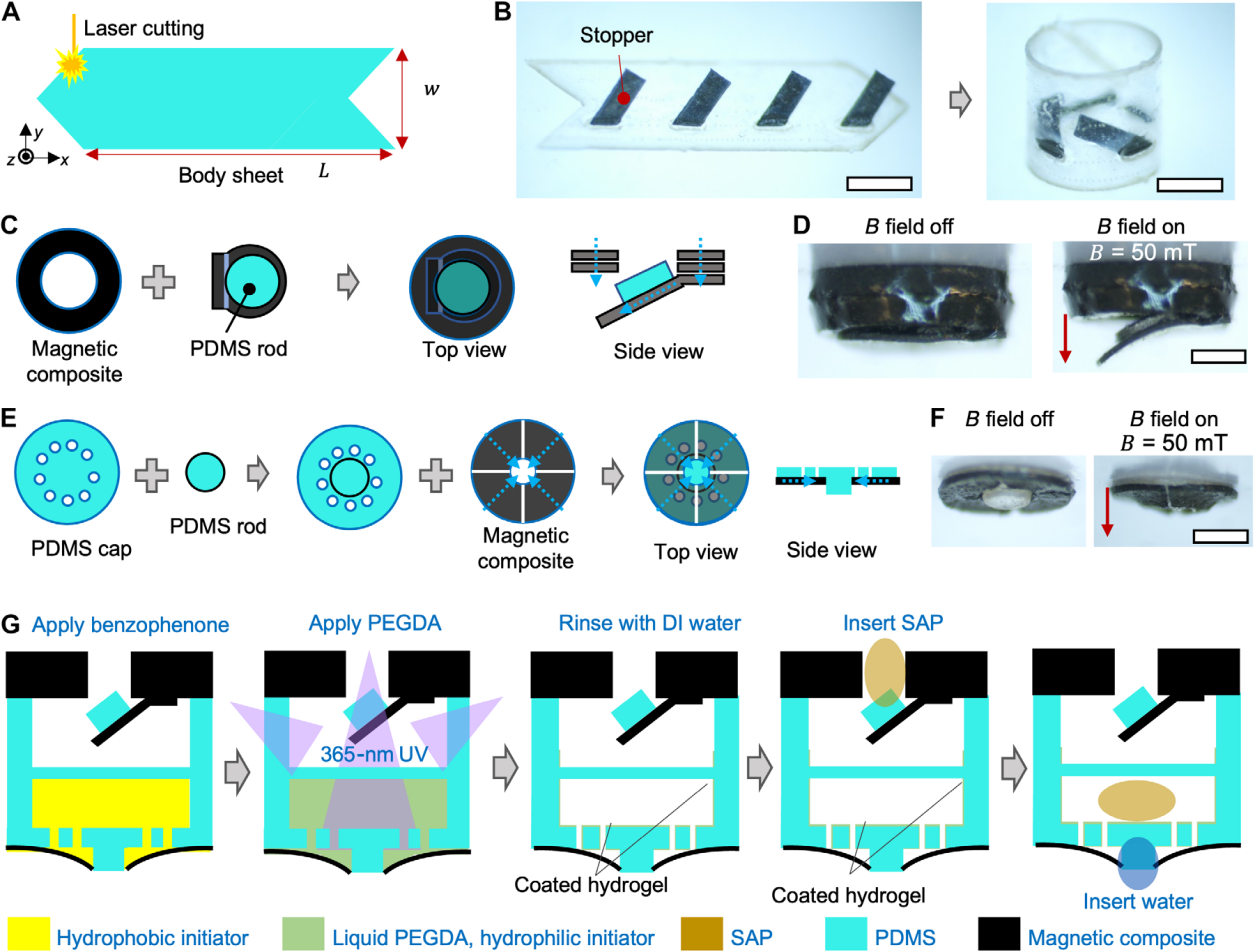
Design and fabrication of the soft capsule body and valve. (**A**) Illustration of the fabrication process of the cylindrical capsule body. (**B**) Image showing thin beams added to the inner surface of the cylinder to constrain the later-interted SAP. Scale bars, 1 mm. (**C**) Illustration of the fabrication process of the capsule outlet valve. The magnetic cap made of magnetic composite (Ecoflex 00-30 and NdFeB microparticles) with a hole is prepared by laser cutting. The magnetization of the assembled outlet valve is also marked with blue dashed arrows. (**D**) Optical images of the outlet valve with or without an external magnetic field applied. *B* = 50 mT. Scale bars, 500 μm. (**E**) Illutration of the fabrication process of the capsule inlet valve. (**F**) Optical images of the inlet valve with or without an external magnetic field applied. *B* = 50 mT. Scale bar, 1 mm. (**G**) Hydrogel integration process including hydrogel coating and the insertion of SAP. PEGDA, poly(ethylene glycol) diacrylate; PDMS, polydimethylsiloxane; DI, deionized.

In Fig. 2, we present the design of the soft capsule and its fabrication process, incorporating laser machining, microassembly, and chemical coating. First, Fig. 2A illustrates the design and fabrication of the capsule body, which is fabricated by laser cutting a PDMS sheet (length: 5.2 mm, width: 1.6 mm, thickness: 120 μm) and subsequently wrapping it into a hollow tube by bonding the two ends under a stereomicroscope. The notch on the PDMS sheet facilitates the alignment during the bonding process. PDMS is chosen due to its biocompatibility (*39*) and transparency for ease of quantifying the sampling process using optical imaging. Figure 2B demonstrates the integration of flexible structures, termed “stopper,” onto the capsule body to confine the SAP in contact with the inlet valve. This constraint is pivotal for absorbing water-based liquids into the capsule body. The stopper is composed of Ecoflex 00-30 and nonmagnetized NdFeB microparticles, chosen for their visibility in x-ray medical imaging, a specific feature to be discussed in the “Soft capsule delivery, sampling, and retrieval under medical imaging” section.

In Fig. 2C, we present the design, magnetization, and assembly of the outlet valve. Without the valving control, the SAP would continuously swell, leading to its extrusion from the capsule. The outlet valve is designed to open when a substantial magnetic field is applied, allowing for the expulsion of air bubbles. To construct the outlet valve, a magnetic ring (diameter: 1.6 mm, thickness: 0.42 mm, hole diameter: 0.60 mm) is formed by stacking multiple thin magnetic rings made of PDMS and NdFeB magnetic particles. A magnetic “leaf” is then affixed to the stacked magnetic ring. As shown in Fig. 2C, the magnetic ring has an axially aligned magnetic moment for steering the capsule by providing a net magnetic moment for the assembled capsule. Meanwhile, the magnetic “leaf” exhibits a radial magnetic moment distribution in-plane, facilitating its opening and closing when subjected to an external magnetic field in the body axial direction. Last, to enhance sealing, a “sealing rod” (diameter: 0.6 mm, height: 0.1 mm) made of PDMS is further bonded to the magnetic leaf, forming a peg-in-the-hole valve. As shown in Fig. 2D, upon the application of a magnetic field (*B* = 50 mT) along the axial direction of the magnetic ring, the magnetic leaf opens with a bending angle of 15°.

Figure 2E illustrates the fabrication process of the inlet valve, crucial for controlling the triggering of water-based liquid sampling. Comprising two components including a cap with multiple holes and a sealing rod, as well as a magnetic membrane, the inlet valve is prepared by cutting a PDMS and magnetic composite sheet. The circular cap features 12 small holes, each with a diameter of 120 mm. The “sealing rod” is then bonded to the circular cap (diameter: 0.6 mm, height: 210 μm). The magnetic membrane, equipped with a hole (diameter: 0.55 mm to 0.7 mm), is a magnetically programmed soft composite structure capable of active bending under applied magnetic fields. To realize the liquid sampling, the holes on the circular cap are designed to be hydrophilic through hydrogel coating. When closed, as shown in Fig. 2F, the circular cap could be covered by the magnetic membrane, sealing the inlet valve. Please see fig. S2 for further details of the magnetization and modeling of the magnetic valves.

To enhance the wetting-based pumping of water-based liquids within the capsule, we introduce a hydrogel coating procedure on the capsule body as shown in Fig. 2G and fig. S3. The inner surface of the body and inlet valve in the assembled capsule undergo a thin layer of hydrogel (thickness: about 10 μm), specifically poly(ethylene glycol) diacrylate (PEGDA) (*40*, *41*) as visualized in fig. S4. SAP modules are subsequently inserted into the capsule housing, ensuring contact with the inlet valve and securing them with the stopper. The hydrogel coating effectively reduces the water contact angle, allowing water-based liquids to be absorbed through the small holes on the cap of the inlet valve. To establish a water channel in the inlet valve, a water droplet is pre-applied on the SAP through the inlet valve.

### Characterization and optimization of soft capsules for controlled triggering and pumping

**Fig. 3.**
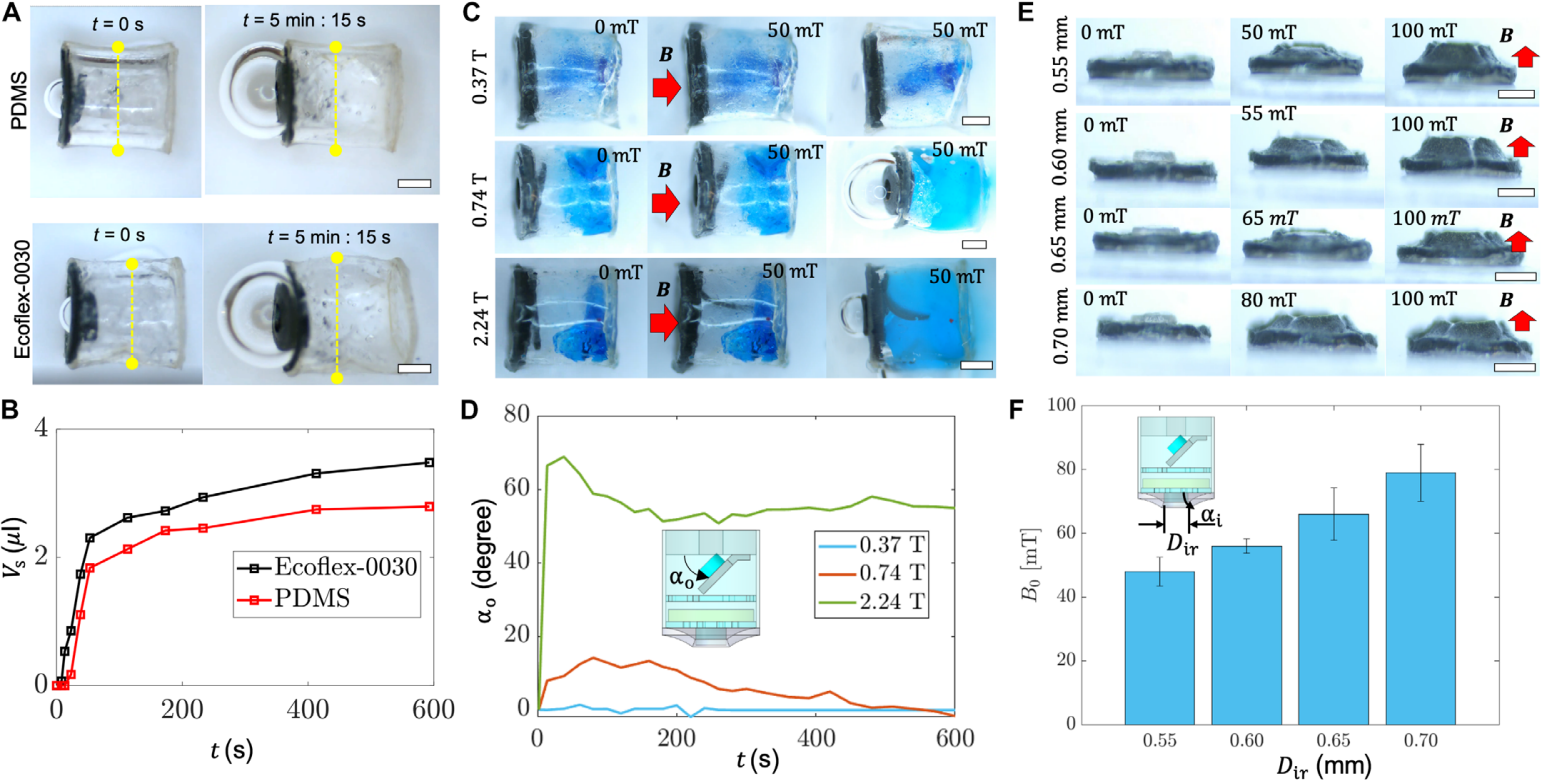
Characterization of the capsule body and valve. (**A**) Snapshots of two soft capsules with PDMS and Ecoflex 00-30 bodies, respectively, integrated with SAP. Scale bars, 500 μm. (**B**) Volume of the sampled liquid (DI water) as a function time for two capsules with different body materials. (**C**) Snapshots of the sampling process for capsules integrated with the outlet valve. A 50-mT magnetic field is applied to open the outlet valve. Scale bar, 500 μm. (**D**) Opening angles of outlet valves with different magnetization over time. (**E**) Optical images of opening of the inlet valves magnetized under different magnetic fields. Scale bars, 500 μm. (**F**) Triggering magnetic field *B*_0_ for opening the inlet valve when varying the sealing rod diameters. Error bar indicates standard deviation for *n* = 5 measurements. The hole diameter of the bonded magnetic leaves is 0.6 mm.

To enhance the design of the capsule for liquid sampling, we systematically characterize the pumping and sealing performance of different soft capsule designs, as shown in Fig. 3. In Fig. 3 (A and B), we explore the impact of the elastic modulus of the body material on sampling behavior. Specifically, we compare a PDMS body with an elastic modulus of about 1.35 MPa to an Ecoflex 00-30 body with an elastic modulus of about 70 kPa. Figure 3B shows the time-dependent volume of the sampled liquid, tracked by monitoring the diameter of the ejected air bubble for capsules with either an Ecoflex 00-30 or a PDMS body house. The PDMS body house exhibits minimal deformation, whereas the Ecoflex 00-30 body house results in a body diameter change with an enlarging factor of 1.2 similar to a balloon (Fig. 3B and fig. S5). While an increased body diameter may enhance the sampling of liquids in terms of volume compared to scenarios with no body deformation, it may also impede the capsule’s ability to navigate small branches of lumens within an organ and increase the risk of blockage. Therefore, a relatively rigid capsule house is preferred, ensuring that the capsule size remains constant before and after sampling liquids, preserving its capability to traverse narrow channels unaffected.

We further refine the design of the outlet valve, aiming for efficient ejection of air bubble and secure sealing together with the swollen SAP as demonstrated in Fig. 3 (C and D). The primary objective is to facilitate effective opening when a substantial magnetic field is applied to allow air ejection during pumping. Meanwhile, the outlet valve should readily close after being pushed by the swollen SAP to ensure a tight seal. In Fig. 3 (C and D), we investigate the opening behaviors of the magnetic “leaves” on the outlet valve, examining different magnetization magnitudes. As shown in Fig. 3 (C and D), when the magnetization is too weak (*M* < 20 kA/m, magnetized in a 0.37-T magnetic field), the magnetic valve may fail to open (opening angle: 10°) due to surface adhesion and friction, rendering the capsule unable to sample any liquids due to the internal air pressure (top row of Fig. 3C). Conversely, when the valve is magnetized in a 2.24-T magnetic field (*M* = 62 kA/m), a 50-mT magnetic field will sustain the valve in an open position, even when the SAP swells, elevating the risk of SAP growing beyond capsule body (bottom row of Fig. 3C). Under these conditions, the valve cannot be pushed to close, even with the pressure generate by the swollen SAP. In contrast, when magnetized in a 0.74-T magnetic field, the valve can be efficiently opened at a bending angle of 15° for effective air bubble ejection and tightly sealed when applying a magnetic field of 50 to 60 mT during the sampling process (middle row of Fig. 3C).

Furthermore, in Fig. 3 (E and F), we further present the characterization of inlet valves with different sealing behaviors, primarily adjusting the ratio between the sealing rod diameter and the magnetic membrane hole diameter to control the critical magnetic field *B*_0_ for valve opening and closing. This ratio governs the prestress between the magnetic membrane and the sealing rod, inducing static friction that maintains a secure seal. When subjected to a relatively small external magnetic field, the valve remains closed due to the static friction between the magnetic membrane and the sealing rod. For simplicity, we maintain the diameter of the hole (*D*_h_= 500 μm) and the height (*h*_s_ = 210 μm) of the sealing rod while varying the sealing rod diameter (0.55 to 0.7 mm). Figure 3 (E and F) reveals that the minimum magnetic field *B*_0_ required to open the valve increases from 42 to 75 mT with the increasing diameter of the sealing rod. A carefully chosen *B*_0_ ensures that the capsule is not triggered during navigation yet remains responsive to the magnetic field generated by the magnetic actuation system.

### Characterization and optimization of liquid sampling performance

**Fig. 4.**
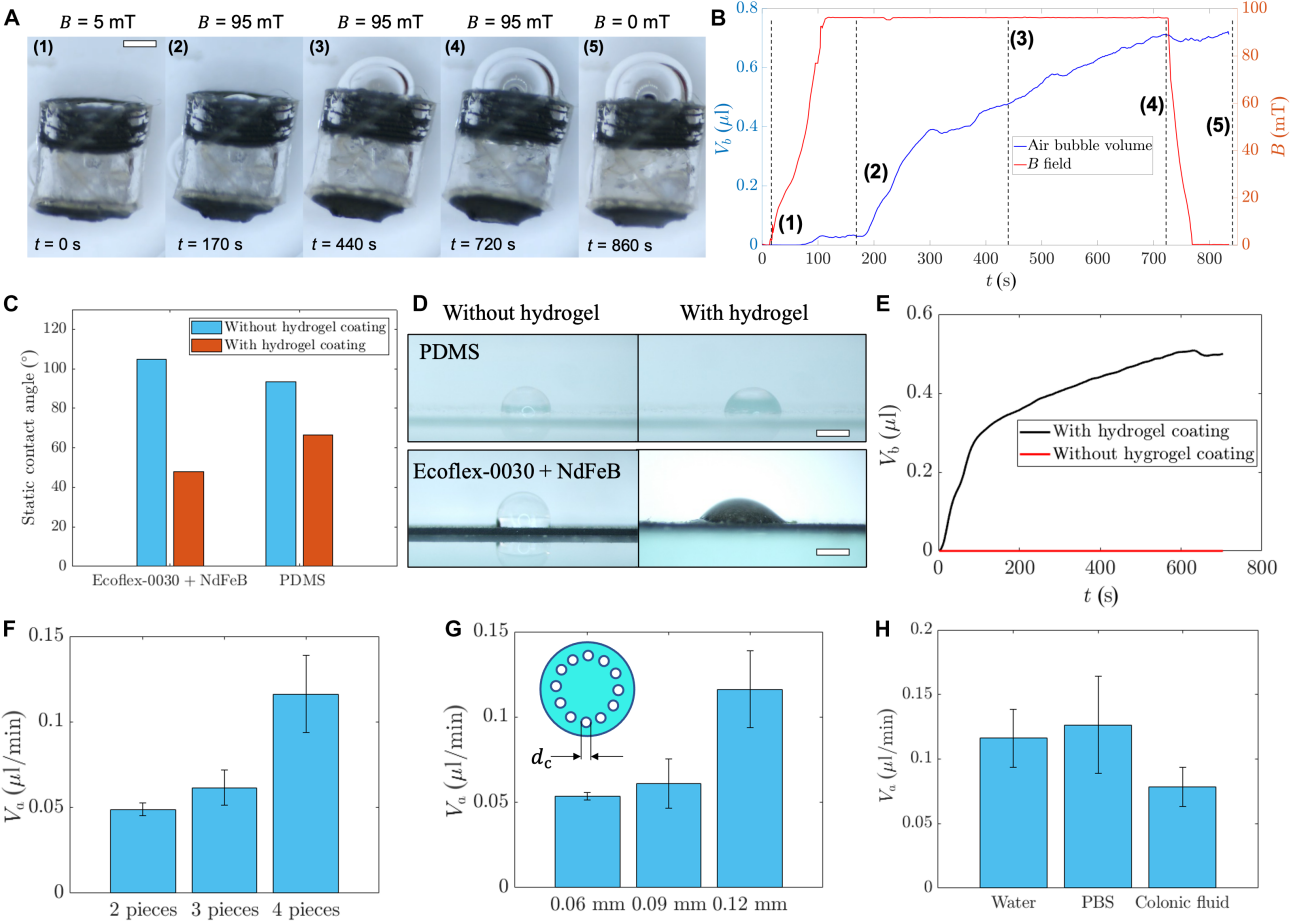
Characterization of the opening, sampling, and sealing process. (**A**) Video frames (movie S1) of a soft capsule sampling water controlled by external magnetic fields. Scale bar, 500 μm. (**B**) Extruded air bubble volume and external magnetic field as a function of time. The marked numbers correspond to the time frames in (A). (**C**) Contact angle of substrates with or without hydrogel coating. (**D**) Optical image of water droplets on surfaces coated with or without hydrogel. Scale bars, 1 mm. (**E**) Average sampling speed of water for soft capsules with or without hydrogel coating on the inlet valve. (**F**) Average sampling speed of water for soft capsules with different integrated SAPs. The average mass of the SAP pieces is 0.147 mg per piece. (**G**) Average sampling speed of DI water for soft capsules with different hole sizes in the inlet valve. (**H**) Average sampling speed of liquids for soft capsules in water, PBS, and colonic fluid. Error bars indicate SD of *n* = 3 trials.

To evaluate the sampling performance, we fabricate a series of soft capsules (approximately 20) with diverse designs and assess their pumping, triggering, and sealing capabilities in various biofluids and synthetic liquids. As illustrated in Fig. 4A, the soft capsules are positioned within the liquid, sinking to the container’s bottom due to their higher overall density. When applying a relatively small magnetic field (<10 mT), both the inlet and outlet valves remain closed, and air remains inside the capsule. A Hall-effect sensor (TLV493D-A1B6-3D Magnetic Sensor, Infineon Inc.) is affixed to the container’s surface to record the external magnetic field. Figure 4A shows a typical sequence involving triggering, sampling, and sealing. Upon increasing the magnetic field magnitude, the inlet valve opens, initiating liquid sampling. The SAP pumps in water-based liquids such as DI water and phosphate-buffered saline (PBS), causing it to swell. As the SAP continues to swell, an air bubble is expelled through the top cap hole, and the volume of the sampled liquid is estimated by tracking the air bubble’s contour and calculating its volume. The whole process takes approximately 10 min, which could be sped up by further optimization of the hole size of the water channels on the inlet valve and the hydrogel surface coating quality. Following the sampling test, the swollen SAP can be retrieved through the top cap hole for future extraction of the sampled liquids (see the “Soft capsule delivery, sampling, and retrieval under medical imaging”).

We further characterize the liquid sampling speed and valve control performance. Figure 4B shows the sampled liquid volume determined by measuring the volume of the ejected air bubble and the magnetic field for controlling the whole sampling process. A triggering magnetic field of *B*_0_ = 90 mT is applied to initiate the sampling process, and the process continues until the outlet valve is closed by the swollen SAP. Upon closure of the outlet valve, the sampling process halts, as the internal pressure is relatively high, preventing further swelling of the SAP. Simultaneously, the inlet valve is closed to ensure a tight seal. To show the necessity of hydrogel coating in Fig. 4 (C and D), we investigate the pumping performance by comparing cases with and without hydrogel coating, when the SAP provides the driving force enabling water to be pumped into the capsule. A comparison is made concerning the water contact angles on the coated or uncoated composite material made by mixing Ecoflex 00-30 and magnetic particles. Figure 4C shows that the water contact angle after hydrogel coating is about 50°, significantly smaller than the angle observed before coating (104°). Coating with hydrogel also demonstrates an enhancement in water wetting ability. In Fig. 4D, the hydrogel coating allows easier wetting and spreading of water on the material surface. Furthermore, Fig. 4E presents the sampled liquid volume as a function of time for miniature capsules with or without hydrogel coatings. Without hydrogel coating, the capsule is unable to pump any fluid inside due to the hydrophobic surface properties trapping air bubbles, as illustrated in Fig. 4E.

Various factors may contribute to the sampling speeds, including the SAP volume, the hole size of the inlet valve, and the type of fluids. First, in Fig. 4F, the average speed of liquid sampling is depicted with different volumes of SAP initially applied inside the soft capsules. A larger amount of SAP insertion results in a higher sampling speed. The time resolved data are shown in fig. S6. Second, as shown in Fig. 4G, the average sampling speed is approximately 0.12 μl · min^−1^ for the capsules with a hole diameter of 120 μm compared to around 0.05 μl · min^−1^ when the hole diameter is 60 μm (fig. S7). Further increasing the hole size should be avoided, as it may lead to SAP leakage from the inlet valve. Last, to quantify the sampling performance across different body fluids, Figure 4H quantifies the sampling performance of the soft capsules in DI water, PBS, and porcine colonic fluids (fig. S8). In comparison to DI water, the sampling speed for the gastric fluids containing more Na^+^ is slightly slower as the sampling speeds for various liquids are influenced by the concentration of the Na^+^ (*42*).

### Control of rolling locomotion of soft capsules in confined spaces

**Fig. 5.**
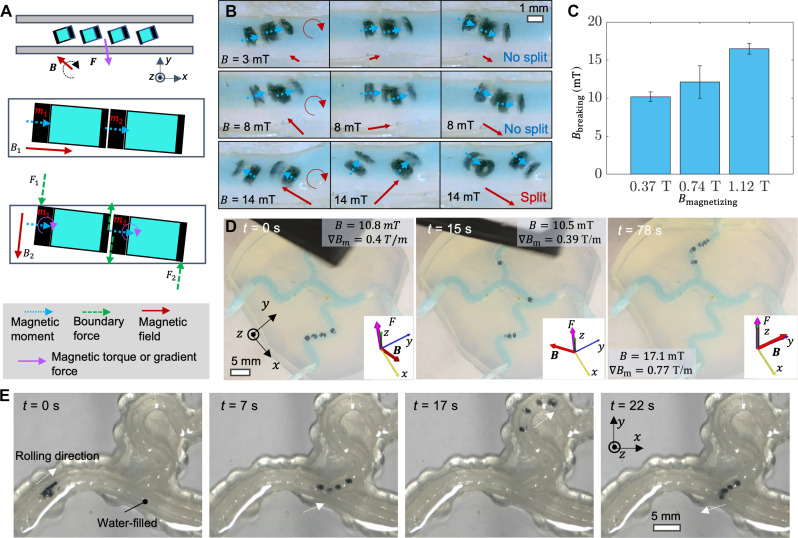
Magnetic control of a self-assembled capsule chain breaking and then rolling motion of the separated capsules inside tube-like structures. (**A**) Illustration of collective motion of four soft capsules in tubular structures actuated by a rotating magnetic field. (**B**) Optical images showing the spliting behavior of the soft capsule chain under varying magnitudes of a rotating magnetic field. (**C**) Critical magnetic field magnitude for breaking the chain of two magnetic capsules magnetized with different magnetizing field magnitudes. Error bars indicate SD for *n* = 5 measurements. (**D**) Optical images of the navigation of three soft capsules inside a phantom. The direction and magnitude of the external magnetic field and force are marked. (**E**) Video frames (movie S2) capturing a group of soft capsules maneuvering within a phantom structure using a rolling locomotion.

It is essential to increase the number of capsules for liquid sampling to ensure a sufficient volume of the liquid sample for about 20 μl based on the current analysis techniques such as droplet digital polymerase chain reaction (ddPCR) (*43*). Consequently, effective coordinated locomotion control for a group of soft capsules becomes crucial for accessing fluid-filled and confined spaces. We propose a strategy for controlling the locomotion of a group of magnetic miniature robots within tortuous tubular structures (*44*–*46*). The group locomotion is achieved by a rotating magnetic field as shown in Fig. 5A. The magnetic field is generated by a permanent magnet mounted on a robotic arm (see fig. S2 for details). The motions are controlled by external magnetic fields, while the magnetic interaction between soft capsules may lead to clustering. To address this issue, these soft capsules are detached from each other using the confinement of the boundary walls to generate a shear force for breaking the clustering at the capsule interface (see the “Analysis of the breaking force for the magnetic capsule chain” section in Materials and Methods for the force analysis). Once separated, the capsules roll on the boundary wall surface facilitated by a magnetic gradient pulling force applied on the capsules for enhanced static friction. In Fig. 5 (B and C), we investigate the clustering effect of the two capsules. A larger magnetic moment requires a larger magnetic field to separate the magnetic chains. Given a specific magnetic moment of the capsules, increasing the magnetic field strength makes it easier for the robots to break the chain but raises the risk of unintentionally triggering the capsule. In addition, we also incorporate the stopper made of polymer and magnetic particles to allow the SAP to be constrained in place for a reliable triggering. The detailed design has been investigated in figs. S9 and S10, suggesting that a medium width and thickness should be used to allow a relatively fast pumping speed and a relatively large total sampled volume.

We demonstrate the controlled navigation of a group of soft capsules within tortuous phantom structures using the mechanism illustrated in Fig. 5D. The magnetic actuation system, as shown in Fig. 5D and fig. S11, relies on permanent magnets mounted on a robotic arm, which comprises two permanent magnets (25 mm by 25 mm by 25 mm, N52, NdFeB) affixed to a stepper motor designed to generate desired spatiotemporally varying magnetic fields. The stepper motor (Nema 17, StepperOnline) is installed on the fringe part of a robotic arm with seven degrees of freedom (Panda Research Package, Franka Emika GmbH). A customized software interface, developed based on the robot operating system, facilitates the control of both the robotic arm and the stepper motor using a joystick (Sony Playstation 4 Dual Shock 4 Controller). Two video cameras (Sony IMX323 USB Camera) monitor the workspace with dimensions of 10 cm by 10 cm by 10 cm, and the robots’ positions are acquired by clicking on the image to inquire about the magnetic field via a calibrated magnetic field model. The magnetic actuation system can provide a magnetic field up to 100 mT and a magnetic field gradient up to 1.5 T/m, with a rotating magnetic field actuation frequency of up to 5 Hz.

In Fig. 5D, the coordinated locomotion of four capsules within a phantom is demonstrated and explained. The magnetic field and gradient pulling force are predicted using a magnetic dipole model and further validated through a magnetic sensor. In a different phantom exhibiting a more meandering structure, the magnetic capsules demonstrate their ability to roll along the inner surface of tubes, gaining access to highly confined and tortuous structures, as shown in Fig. 5E and movie S2. The soft capsules have been shown to perform a rolling locomotion but could be further combined with miniature soft robots with other locomotion modes such as swimming (*47*), crawling, and climbing (*25*, *27*), for navigating diverse environments. This integration could enable these soft capsules to navigate through even more complex and challenging environments.

### Soft capsule delivery, sampling, and retrieval under medical imaging

**Fig. 6.**
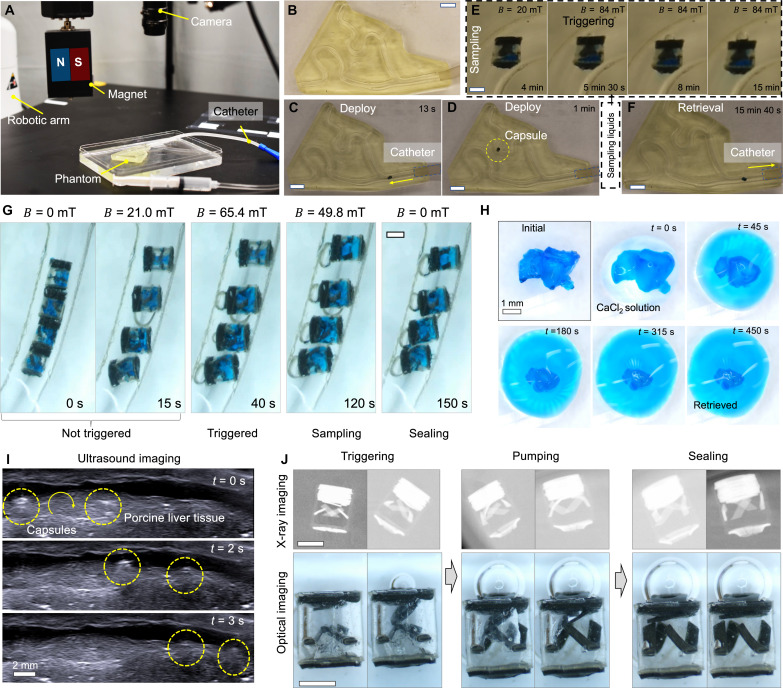
Medical imaging-guided locomotion and liquid sampling demonstration by a soft capsule. (**A**) Experimental setup demonstrating the deployment and retrieval of the capsule using a catheter. (**B**) Image of the 3D printed phantom. Scale bar, 5 mm. (**C**) Video snapshot (movie S2) of delivery of the capsule through a catheter. Scale bar, 5 mm. (**D**) Video snapshot (movie S2) of the capsule locomotion inside the phantom. Scale bar, 5 mm. (**E**) Video snapshot (movie S2) of the sampling process for the soft capsule. Scale bar, 1 mm. (**F**) Video snapshot (movie S2) of the retrieval of the soft capsule by a catheter. Scale bar, 5 mm. (**G**) Video snapshot (movie S2) of a group soft capsules sampling in a phantom. Scale bar, 1 mm. (**H**) Optical images of retrieving the sample liquids using a calcium chloride solution. (**I**) Video frames (movie S3) of a soft capsule rolling in a porcine bile duct visualized using an ultrasound imaging machine. (**J**) X-ray and optical imaging frames of a soft capsule sampling inside a porcine bile duct. Scale bars, 5 mm.

To facilitate the rapid and targeted collection of samples for analysis, steerable flexible catheters (*48*) or centimeter-scale endoscope capsules (*49*) can be used for delivering and retrieving the millimeter-scale capsules for sampling liquids in the GI tract. First, as a proof of concept, we demonstrate the delivery of the capsule inside a phantom using a 14 FR flexible catheter (25 cm in length, 4.7 mm in diameter; Enema Catheter Colon Tube) as shown in Fig. 6A. The experimental setup comprises a permanent magnet actuation system, a three-dimensional (3D) printed phantom, and a flexible catheter. Designed to emulate the structure of the bile duct, the phantom is shown in Fig. 6B. The entire process of capsule delivery, liquid sampling, and capsule retrieval inside the 3D printed phantom is captured in Fig. 6 (C to F). Initially, the flexible catheter reaches the phantom’s inlet but is hindered from further entry due to its size. Subsequently, the millimeter-scale capsule is injected (Fig. 6C) and maneuvered to roll inside the phantom using a rotating magnetic field of approximately 20 mT (Fig. 6D). Upon reaching the targeted location, a larger magnetic field of 84 mT is applied to initiate liquid sampling (Fig. 6E). After approximately 10 min, the capsule rolls back and is retrieved by the flexible catheter (Fig. 6F). In comparison with existing soft capsule robots (*50*, *51*), the proposed millimeter soft capsule integrates valves for controlling the sampling process and exhibits the capability to access more confined spaces when deployed as a group through controlled rolling motion. We further demonstrate simultaneous liquid sampling by four capsules in an open workspace (fig. S12) and within a tubular structure (Fig. 6G). In an open space, these capsules can sample liquids simultaneously, controlled by the same external magnetic field, with their positions fixed. With no fixed positions, a group of soft capsules form a chain due to magnetic interaction and remain inactive when a relatively small magnetic field is applied. In contrast, when the four capsules are placed inside a phantom tube with an inner diameter of about 2.8 mm, they initially form a chain when no magnetic field is applied or when the applied magnetic field is relatively weak. A magnetic field of 21 mT can break the capsule chain when the field is perpendicular to the channel. The capsules then begin sampling when the magnetic field reaches approximately 65 mT, completing the process in about 3 min.

Second, we demonstrate the retrieval of the sample from the SAP matrix for subsequent analysis by using calcium chloride solution (*38*). Figure 6H illustrates the retrieval process of the liquids contained within the swelled SAP matrix. Initially, the sample is in a solid state within the SAP matrix. The application of calcium chloride solution induces a rapid shrinkage of the solid sample, releasing liquid samples that can be further analyzed. We can use ddPCR (*43*) for analysis, which is a state-of-the-art method for performing digital PCR based on massive sample partitioning using water-oil emulsion droplet technology. In addition, next-generation sequencing (*52*) can be used for analyzing the microbiome in body fluids, enabling the identification of unique microbiome species without relying on traditional culture methods (*53*). These applications are beyond the scope of this work but will be explored in the future.

Last, to demonstrate the device’s efficacy in navigating fluid-filled and confined tubular structures within animal models, ultrasound imaging is used to guide the robot’s navigation within porcine organs. Porcine liver, procured from a local slaughterhouse in Nashville, TN, USA, is initially refrigerated before being used to assess the locomotion capabilities of the soft capsules within lumens. Ultrasound imaging (ACUSON NX3 Diagnostic Ultrasound System, transducer model: VF 10-5, B-mode, operating frequency: 8 MHz) is used to monitor the robot’s position and orientation in the ex vivo porcine liver, as shown in Fig. 6I and movie S3. In this experiment, a magnetic actuation system featuring two permanent magnets mounted on a hand drill (Cordless Drill, Black+Decker) is used for the convenient manual control of the magnetic field, which could be automated in the future work using the robotic arm-based system.

While ultrasound imaging enables noninvasive tracking of the capsule’s position, the sampling process may not be visualized due to the relatively low resolution. To ensure a clear and precise monitoring of the sampling process, as illustrated in Fig. 6J, we propose to use the x-ray medical imaging. As a proof of concept, an x-ray cabinet imaging system (Faxitron LX-60 x-ray system) is used, which offers a relatively fine spatial resolution (approximately 10 lp/mm). The liquid sampling process is observed by visualizing the deformation of the soft capsule valves and body with integrated deformable markers. To enhance the visualization of SAP swelling behavior, the flexible “stopper” structure is designed with high-density particles (nonmagnetized NdFeB) embedded inside elastomer to distinctly indicate the various swelling states of the capsule. The illustration of the stopper deformation by the SAP is shown in fig. S13. As shown in Fig. 6J, the stopper structures are tilted at an angle of about 45°, aligning nearly at 90° to the wall when the SAP completes swelling, and the stopper structures are pushed against the capsule wall.

## DISCUSSION

In summary, we report millimeter-scale soft capsules for targeted and local liquid sampling within fluid-filled and confined spaces. These capsules, characterized by their small size, are fully controlled using external magnetic fields remotely to allow on-demand sampling and sealing as well as group rolling locomotion. Our approach leverages SAP and magnetically controlled flexible valves to enable efficient sampling of water-based body fluids into the capsules. We have systematically investigated the pumping and valving mechanisms, showcasing their effectiveness in sampling various liquids, including DI water, PBS, and gastric fluids, in vitro. Moreover, we have also presented a mechanism for coordinating a group of soft capsules to navigate inside phantom structures, a task challenging for a flexible catheter. We have showcased the control of a group soft capsules inside fluid-filled and tortuous spaces as well as inside porcine organs ex vivo under the guidance of ultrasound imaging. The group locomotion control allows increasing the sampled volume and system redundancy. Last, our experiments involving medical imaging-based guidance and retrieval demonstrate the potential for future medical operations within organs.

Enhancements to the soft capsules can be explored in several aspects. First, further reduction in capsule size could enable access to even more confined spaces. To reduce the capsule size (current version has diameter of 1.8 mm and a height of 1.6 mm), advanced fabrication techniques such as photolithography, transfer molding, and layer-by-layer integration processes may be used for submillimeter scale capsules. Second, testing these capsules in the mice intestine, porcine bile duct, and pancreatic duct is necessary to further validate their performance comprehensively for in vivo applications. Third, it is essential to note that using soft robotic capsules in blood vessels may pose challenges due to the ejection of relatively large air bubbles (*54*). However, by minimizing air bubble size and incorporating biocompatible oil-based emulsions as substitutes, it may be plausible to conduct liquid biopsy in blood vessels. Careful evaluation of the potential risk of vessel blockage is imperative. In addition, we aim at using SAP for sampling body fluid. Different biomolecules could be targeted by exploring SAP of different porous structure sizes. Literature has shown that SAP can absorb microbiota (*15*) or other biomolecules (*55*) depending on the pore size (*56*). Last, looking ahead, integrating the proposed soft robotic capsules with more complex microfluidic channels and valves could facilitate biomarker sampling at different locations. The millimeter-scale soft capsules introduced in this work thus open avenues for minimally invasive and targeted liquid biopsy, enabling early disease diagnosis and providing insights into disease development through the sampling, retrieval, and analysis of abundant chemicals within organs.

## MATERIALS AND METHODS

### Fabrication of capsule body

The soft modules used for assembling the soft capsules were prepared through a combination of procedures including laser lamination and assembly techniques. The soft elastomer constituting the body is composed of PDMS (with a monomer and crosslinker ratio of 10:1 by weight, Dow Silicones Corporation). To achieve the desired PDMS sheet thickness, glass slides with spacers were used in the fabrication process. The PDMS body sheet was precisely cut using a laser cutter. Subsequently, four stoppers made of SAP were affixed to the inner surface using Ecoflex 00-30 before the body sheet was curled to form a cylindrical shape, securing the two ends together with Ecoflex 00-30 (Smooth-On Inc.).

### Fabrication of capsule outlet valve

The outlet valve was made of NdFeB particles (Magnequench Int.) and PDMS in a 2:1 weight ratio. The magnetic PDMS was cured on glass slides with spacers, resulting in a thickness of 210 μm. The outlet cap was assembled by bonding two layers of magnetic PDMS sheets with Ecoflex 00-30. Each outlet cap sheet featured a laser-cut hole in the center with a diameter of 1 mm. The outlet cap underwent magnetization along its height, achieved using an impulse magnetizer generating a field of 1.8 T. The valve within the outlet cap comprised a leaf and base, both constructed from magnetic PDMS. A hinge was laminated between the leaf and base, enabling the valve to remain closed without the influence of an external magnetic field, owing to predeformation. The valve was magnetized along the normal direction of the long edge of the base. A PDMS rod was affixed to the valve to act as a plug for sealing, and the valve was securely bonded onto the cap using Ecoflex 00-30. Opening the valve could be facilitated by applying an external magnetic field, allowing the expulsion of air bubbles (fig. S13).

### Fabrication of capsule inlet valve

The inlet valve of the soft capsule consisted of two layers with one layer serving as the inlet valve cap, while the other functioning as the magnetic membrane. The top layer was made from PDMS with a thickness of 120 mm. The magnetic membrane was composed of Ecoflex 00-30 mixed with NdFeB particles. This layer features four leaves, with their magnetic moments all pointing toward the center, enabling magnetic attraction to the magnetic valve. The PDMS layer incorporated 12 intricately patterned through-holes, each measuring 120 μm in diameter. In addition, a PDMS sealing rod (with a diameter of 600 μm) was securely bonded at the center of the PDMS layer to introduce prestress, ensuring that the magnetic valve remains closed even with the application of a relatively small magnetic field. The valve can be opened by applying a magnetic field in the body axis direction.

### Integrating hydrogel in the capsule

For the polymer sheet coating process, a benzophenone solution [20 weight % (wt %) in water, Sigma-Aldrich Inc.] was carefully pipetted into the capsule through the outlet valve. This served as the hydrophobic photo-initiator, initiating surface bonding to hydrogels, and was subsequently dried after a 2-min interval. A solution consisting of PEGDA (Sigma-Aldrich Inc.) and DI water at 20 wt % was prepared, incorporating α-ketoglutaric acid (Chem-Impex Intl Inc.) at 1 wt %. This solution was then pipetted into the capsule, facilitated by the opening of the inlet valve through an external magnetic field. The mixture was cured using an ultraviolet (UV) lamp (15 W) for a duration of 30 min, allowing the hydrogel to crosslink on the surface of the inlet valve cap. Upon completion of the coating process, a superabsorbent polymer was introduced into the capsule body through the large hole on the top cap. Approximately 1 μl of DI water was added through the inlet valve to facilitate the swelling of the SSAP (sodium polyacrylate, Sigma-Aldrich Inc.) and fill the space between the SAP stoppers and bottom valve, ensuring complete contact with the hydrophilic surface of the inlet valve.

### Preparation of organ phantoms

The phantom used in Fig. 5D to emulate bile ducts was crafted from Ecoflex 00-31 (Smooth-On Inc.) using a sacrificial molding method. Using a PLA 3D printer, 3D printed structures were used to create a negative mold made of silicone rubber Mold Max 60. Caramel was melted and molded into the desired structures using this negative mold. Subsequently, the caramel structures underwent further molding through casting with Ecoflex 00-31, chosen for its relatively good optical transparency. After the curing of Ecoflex 00-31, the caramel structure was dissolved in hot water. In addition, the phantoms in Figs. 5E and 6B, C, D, and F were directly printed using UV-curable resin in a stereolithography printer (Form 3+, Formlabs Inc.).

### Analysis of the breaking force for the magnetic capsule chain

To investigate the behavior of breaking the capsule chain, we model the simplest case with two magnetic capsules forming a chain as the magnetic attractive force is inversely proportional to *r*^4^, where *r* is the distance between the two magnetic capsules (fig. S14). Each magnetic capsule has a magnetic moment of ***m***_**i**_ (*i* = 1,2), which is linearly dependent on the magnetization and volume of the magnetic capsule. Two magnetic capsules have magnetic interaction torque and force. The magnetic dipole-dipole interactive force ***F***_12_ is given by the following formula

$$\begin{array}{c} F_{12}=\frac{3\mu_{0}}{4\pi {\left|r\right|}^{4}}\left\{\left(\hat{r}\times m_{1}\right)\times m_{2}+\left(\hat{r}\times m_{2}\right)\times m_{1}-2\hat{r}\left(m_{1}\cdot m_{2}\right)\right\}\\ +5\hat{r}\left[\left(\hat{r}\times m_{1}\right)\cdot \left(\hat{r}\times m_{2}\right)\right] \end{array}$$ (1)

where ***r*=*r***_**2**_**−*r***_**1**_ is the displacement vector between the two capsules with magnetic moments ***m***_1_ and ***m***_2_ and μ_0_ is the magnetic permeability of vacuum. $\hat{r}=r/|r|$ is the unit vector and |***r***| **=***h*_b_ is the capsule body height. Before the capsule chain breaks, ***r*** is parallel to ***m***_1_ and ***m***_2_. Assuming that $m_{1}=m_{2}=\mathrm{MV}\;\hat{r}$ , where *M* and *V* are the magnetization and volume of the magnetic capsule, we have

$$F_{12}=-\frac{3\mu_{0}M^{2}V^{2}}{2\pi r^{4}}\hat{r}$$ (2)

where the negative sign represents attractive force. To break the capsule chain, the magnetic torque applied on the capsule body needs to induce a boundary force couple that could overcome the static friction (friction coefficient: µ_s_) due to the magnetic attraction, given by

$$\tau_{m}=\mathrm{MVB}>F_{N}\mu_{s}\cdot h_{b}=\frac{3\mu_{s}\mu_{0}M^{2}V^{2}}{2\pi r^{4}}r$$ (3)

On the basis of Eq. 3, the critical magnetic field *B*_0_ to break the capsule chain is given by

$$B_{0}=\frac{3\mu_{0}\mathrm{MV}}{2\pi r^{3}}$$ (4)
