# Supplementary material for: Millimeter-scale soft capsules for sampling liquids in fluid-filled confined spaces
Source: Sci Adv. 2024 Aug 28;10(35):eadp2758. doi: 10.1126/sciadv.adp2758 (PMC11352903; doi:10.1126/sciadv.adp2758)
Supplement: Supplementary file 1 — Figs. S1 and S14 Legend for movies S1 to S3 [file sciadv.adp2758_sm.pdf]

Science Advances
AAAS

Supplementary Materials for

Millimeter-scale soft capsules for sampling liquids in fluid-filled confined spaces

Xiaoguang Dong *et al.*

Corresponding author: Xiaoguang Dong, xiaoguang.dong@vanderbilt.edu; Metin Sitti, sitti@is.mpg.de

Sci. Adv. **10**, eadp2758 (2024)
DOI: 10.1126/sciadv.adp2758

The PDF file includes:

Figs. S1 and S14
Legends for movies S1 to S3

Other Supplementary Material for this manuscript includes the following:

Movies S1 to S3

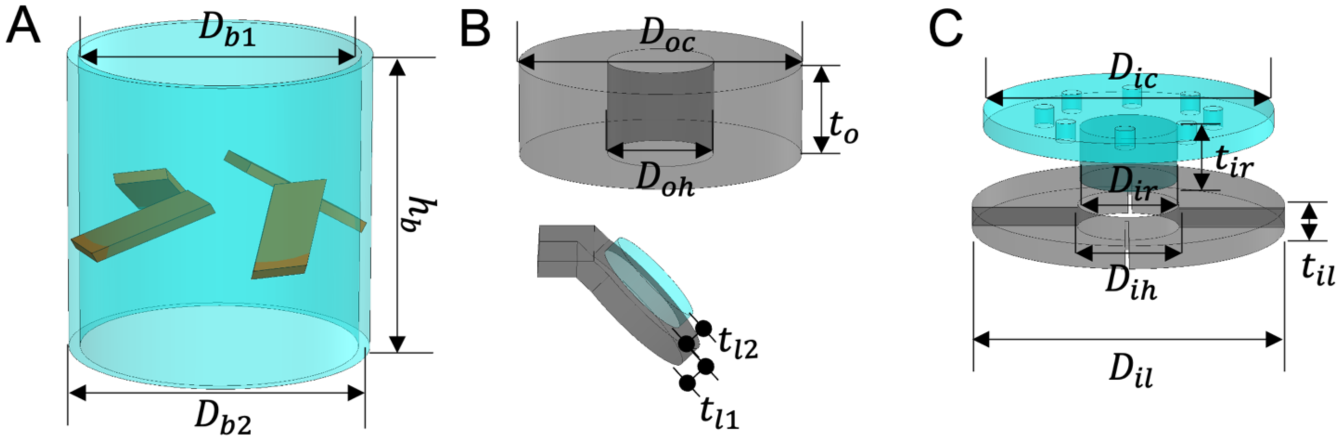

Fig. S1. Dimensions of the soft capsule body, inlet valve, outlet valve. A. Dimensions of the capsule body. Four beam markers made of non-magnetized magnetic composite are bonded to the body for X-ray imaging. Blue color indicates non-magnetic polymer such as pure PDMS. B. Dimension of the outlet valve. Gray color indicates magnetized magnetic composite. C. Dimension of the inlet valve. The number of holes in the PDMS cap is from 8 to 12. In A-C, example capsule parameters, $D_{b1} = 1.55\ mm$, $D_{b2} = 1.6\ mm$, $h_b = 1.8\ mm$; $D_{oc} = 1.55\ mm$, $D_{0h} = 0.5\ mm$, $D_{0c} = 1.55\ mm$, $t_o = 0.5\ mm$, $t_{l1} = 0.25\ mm$, $t_{l2} = 0.25\ mm$; $D_{ic} = 1.6\ mm$, $D_{ir} = 0.6\ mm$, $D_{ih} = 0.9D_{ir}$, $D_{il} = 1.6\ mm$, $t_{ir} = 0.21\ mm$, $t_{il} = 0.15\ mm$.

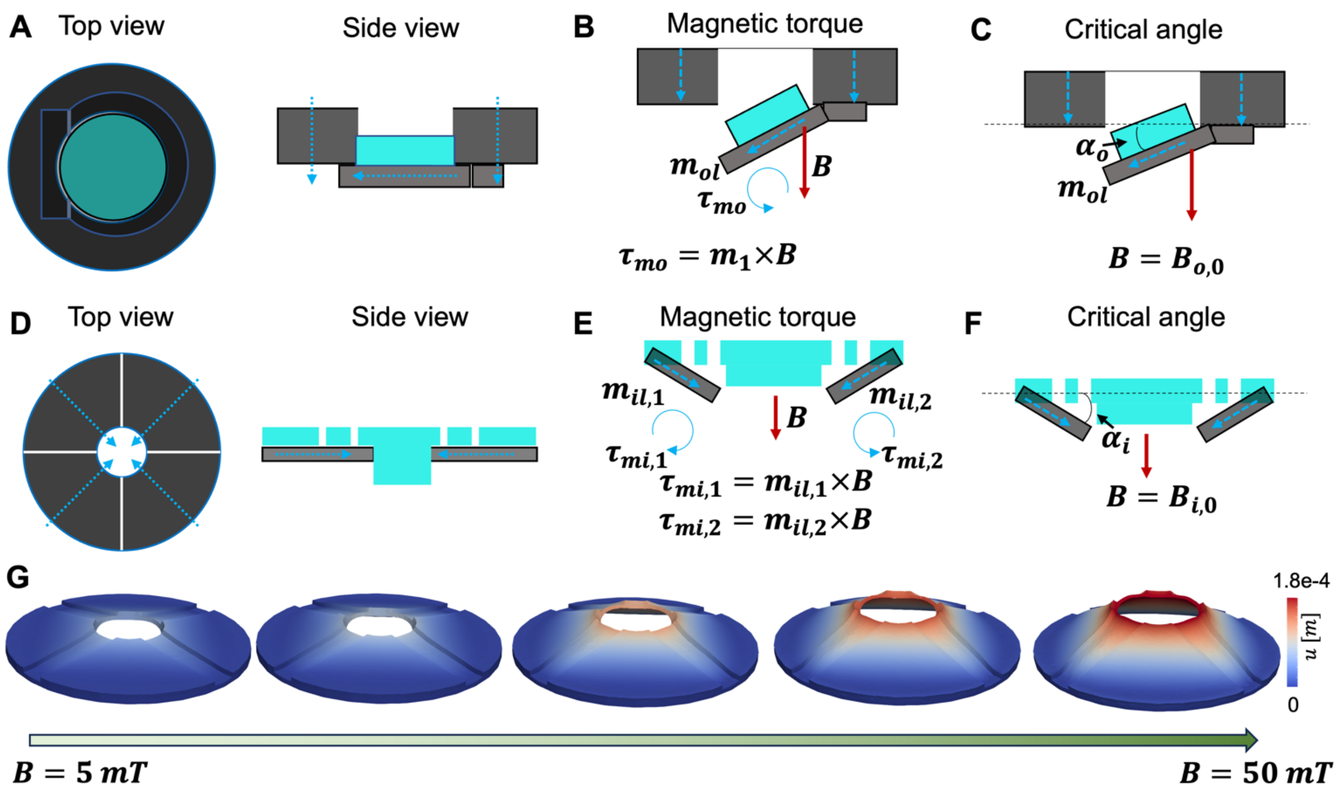

Fig. S2 Modeling of the magnetic valve opening and closing. A. Illustration of the magnetization of the outlet valve. B. Magnetic torques applied on the outlet valve. C. Illustration of the critical angle of the outlet valve when there is space between the hole and the sealing rod. D. Illustration of the magnetization of the inlet valve. E. Magnetic torques applied on the inlet valve. F. Illustration of the critical angle of the inlet valve when there is gap between the magnetic leaves and the sealing rod. G. Finite Element Method based modeling of the inlet valve.

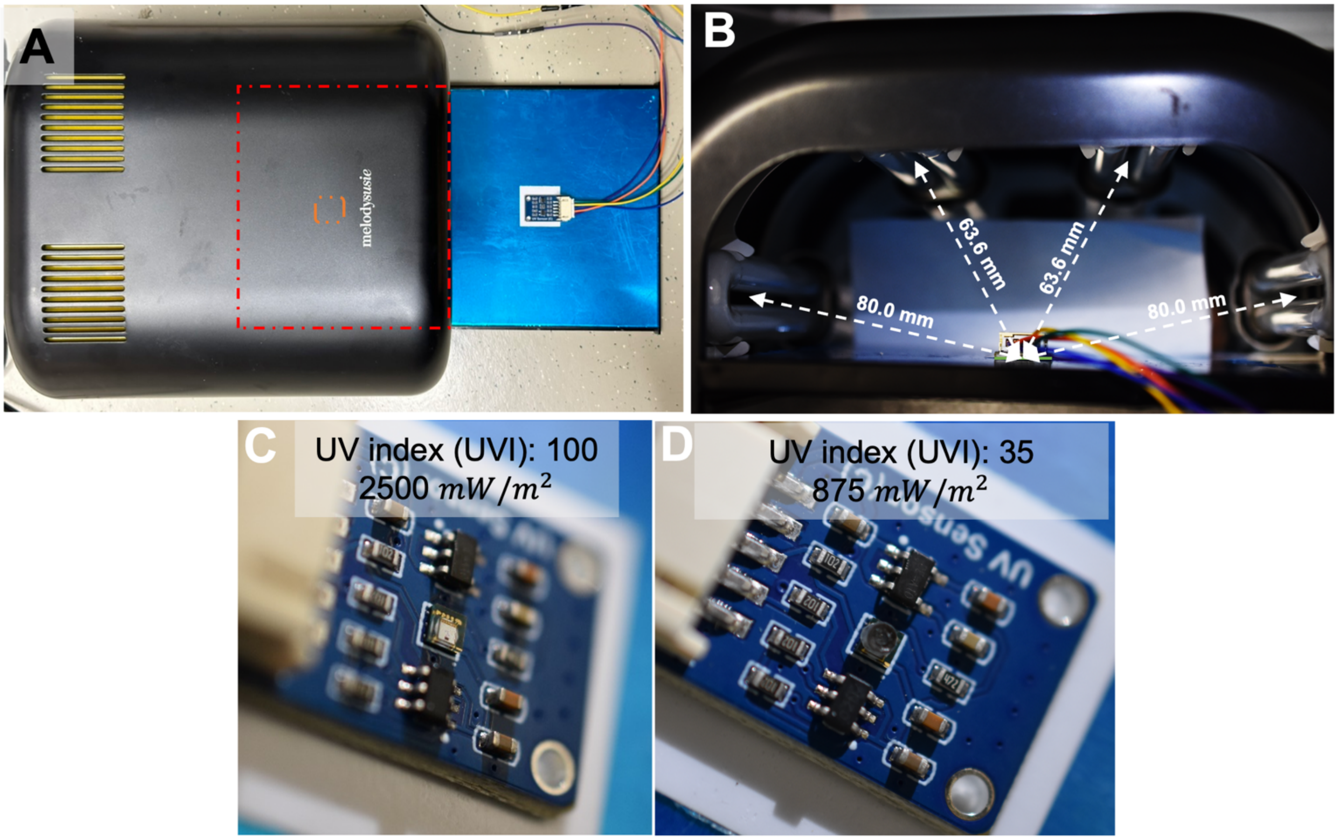

Fig. S3 Characterization of the UV exposure intensity during hydrogel coating. A. Top view of the UV chamber and a UV intensity sensor. B. Side view of the UV chamber and a UV intensity sensor with marked distances. C. Optical image of the UV intensity sensor without a capsule covering the sensor. UVI: 100. D. Optical image of the UV intensity sensor without a capsule covering the sensor. UVI: 35.

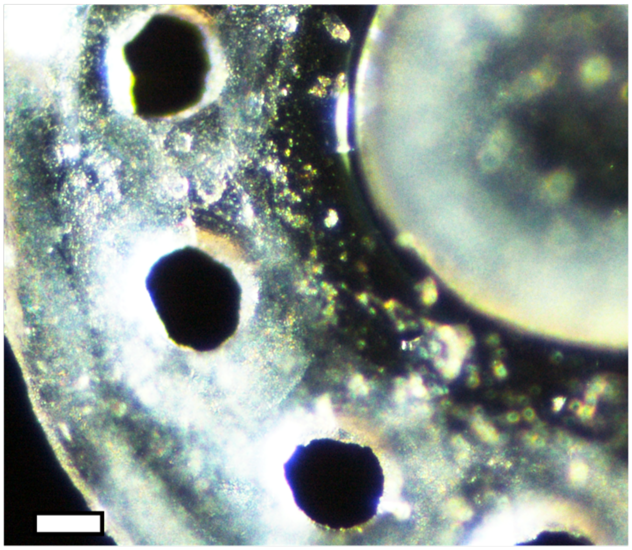

Fig. S4 Characterization of the hydrogel coating. Scale bar, 90 μm.

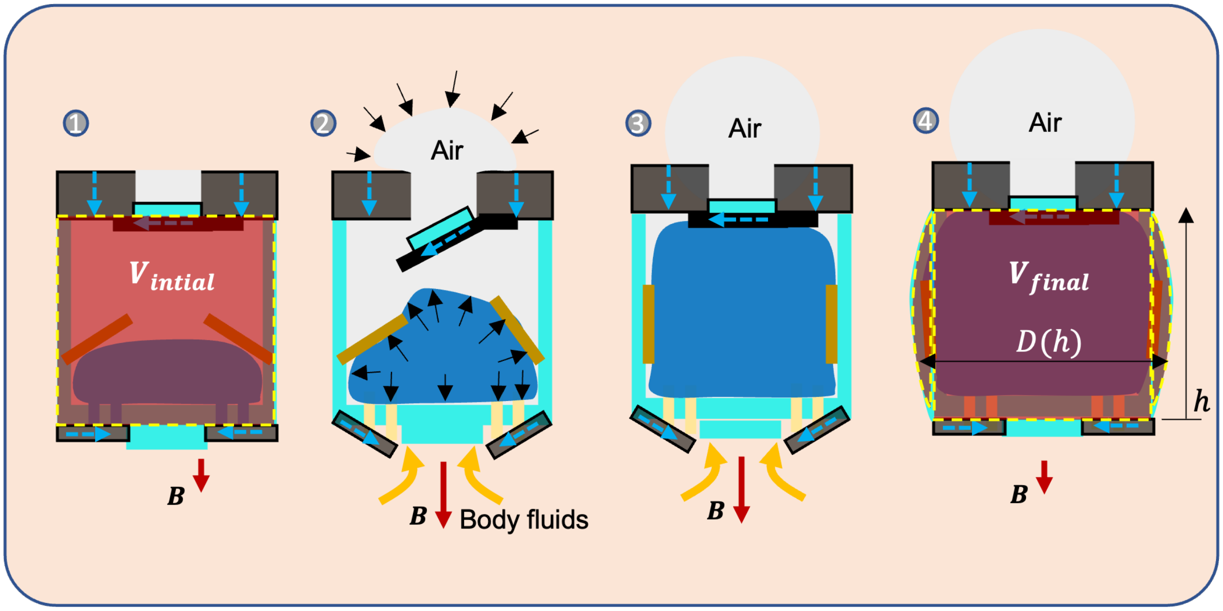

Fig. S5 Illustration of the inflation of the soft body of the capsule.

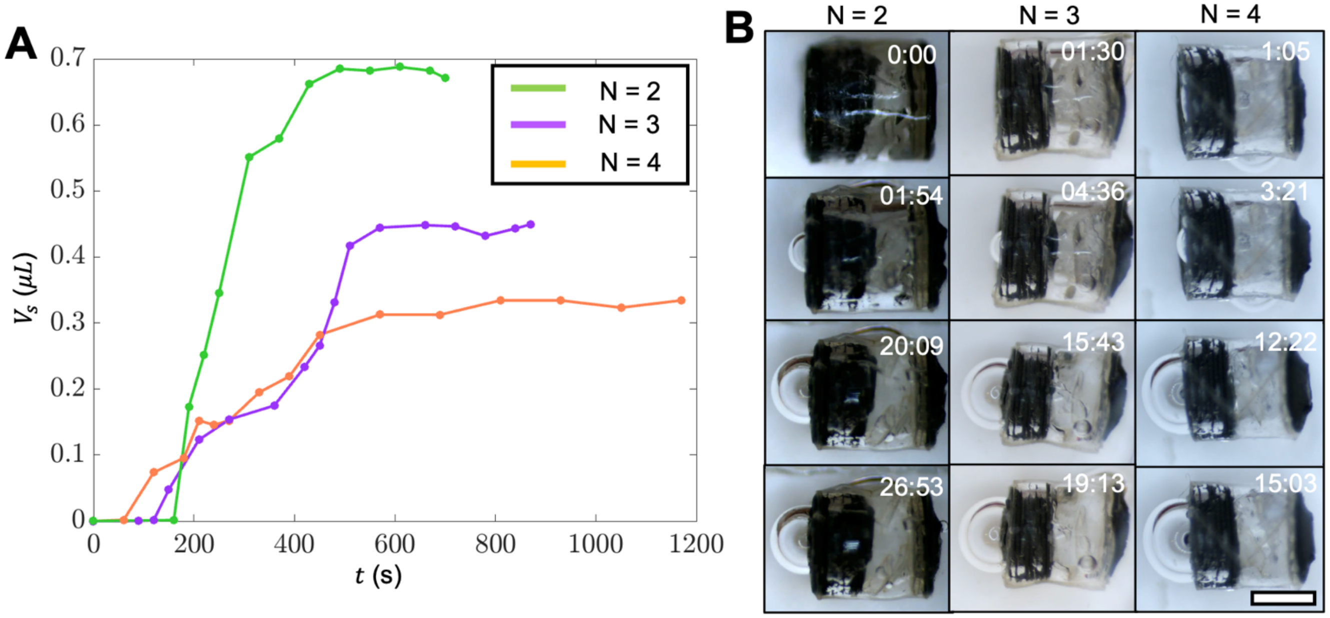

Fig. S6 Characterization of sampling water by soft capsules with different SAPs. A. Sampled liquid volume as a function of time for capsules with N = 2, 3 and 4 pieces of SAPs. B. Video frames of sampling liquid by soft capsules with different SAPs. Time stamp format, minutes : seconds. Scale bar, 1 mm.

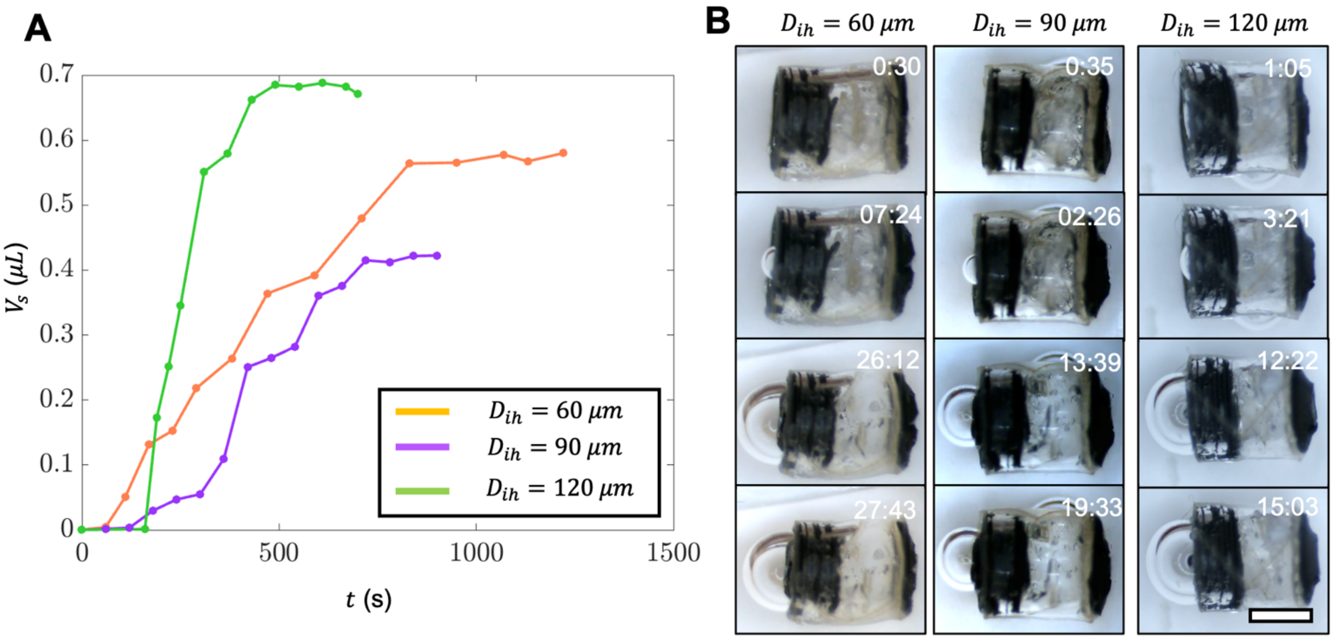

Fig. S7 Characterization of sampling water by soft capsules with different inlet valve hole diameters. A. Sampled liquid volume as a function of time for capsules with different inlet valve hole diameters. B. Video frames of sampling liquid by different soft capsules. Time stamp format, minutes : seconds. Scale bar, 1 mm.

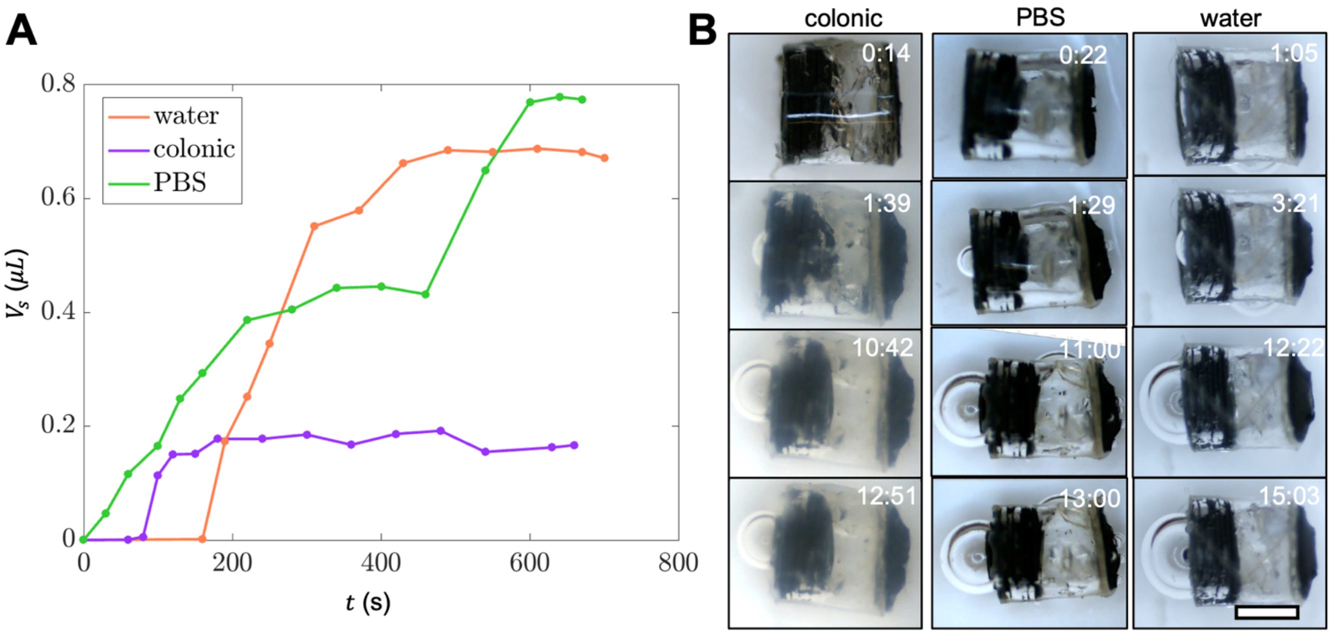

Fig. S8 Characterization of sampling different fluids by a soft capsule. A. Sampled liquid volume as a function of time for a soft capsule in different liquids. B. Video frames of sampling liquid by the soft capsule. Time stamp format, minutes : seconds. Scale bar, 1 mm.

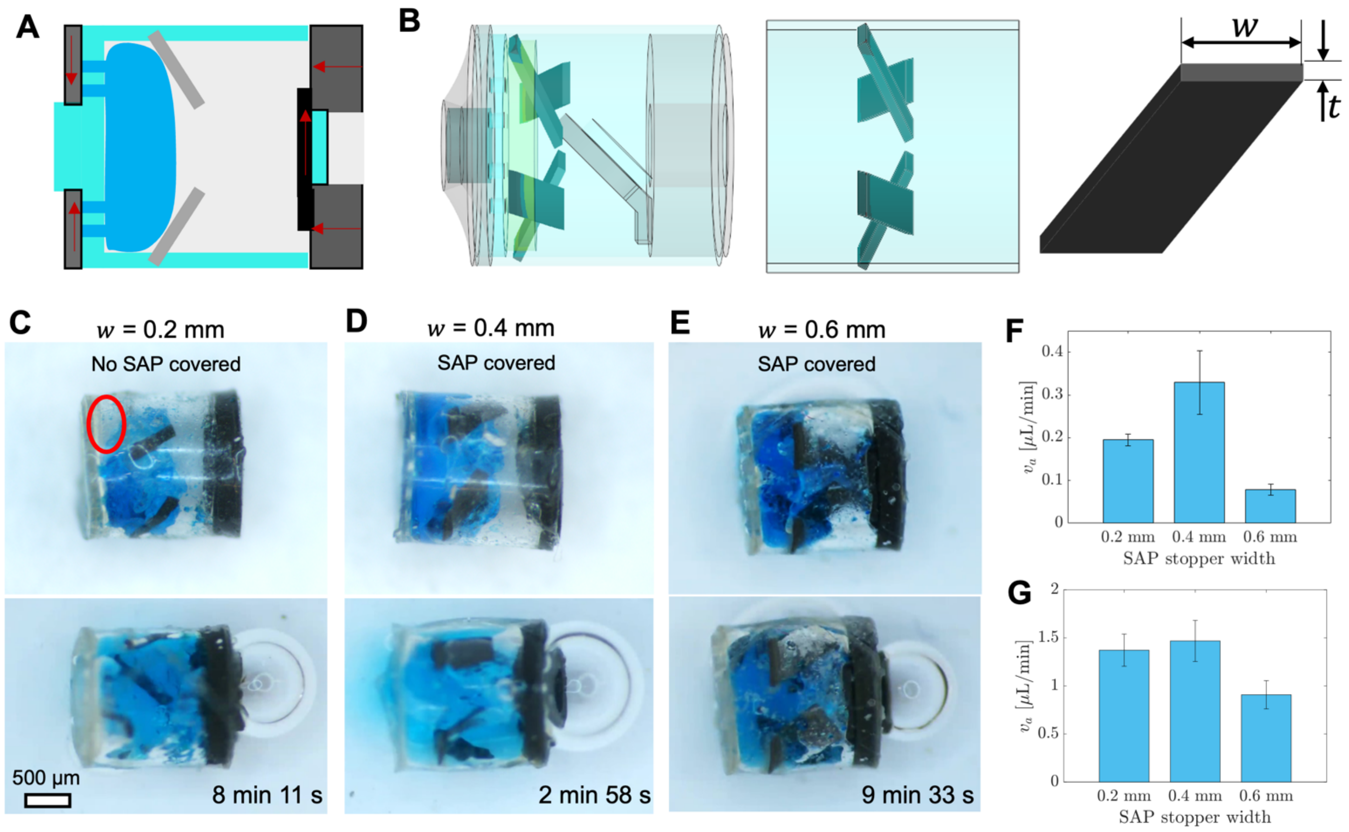

Fig. S9 Design of the stopper width for optimizing the sampling performance. A. Illustration of the stopper function. B. Illustration of the stopper geometric parameters. C-E. Optical images of the initial and final states of the capsules with different stopper designs including (C) w = 0.2 mm, (D) w = 0.4 mm, (E) w = 0.6 mm. F. Average sampling speeds for capsules with different stopper widths. G. Total sampled volume for capsules with different stopper widths.

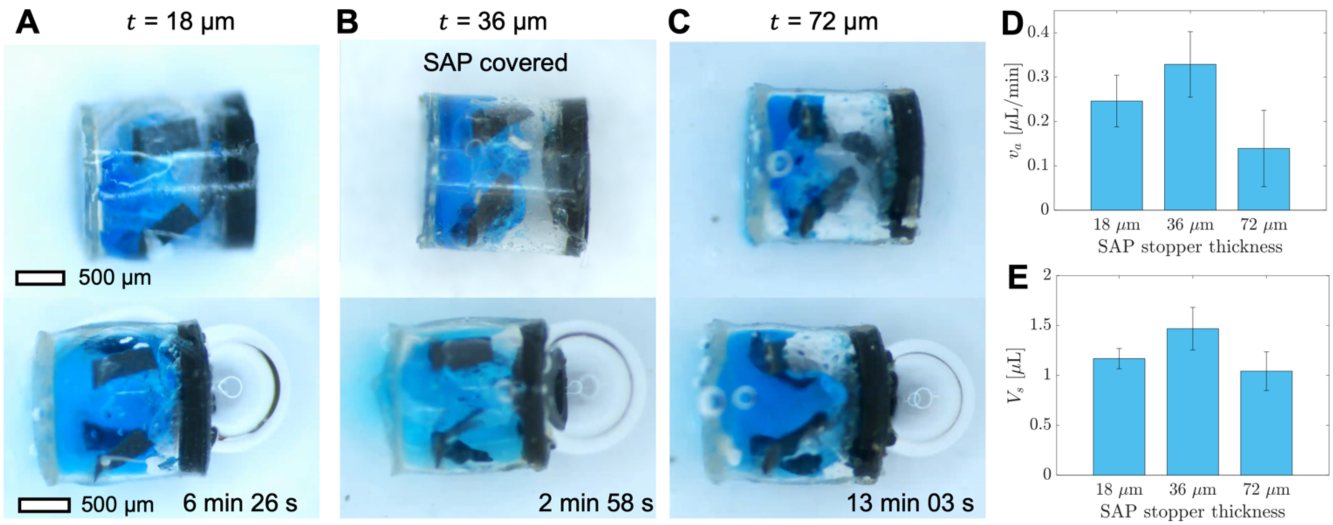

Fig. S10 Design of the stopper thickness for optimizing the sampling performance. A-C. Optical images of the initial and final states of the capsules with different stopper designs including (A) t = 18 μm, (B) t = 36 μm, (C) t = 72 μm. D. Average sampling speeds for capsules with different stopper widths. E. Total sampled volume for capsules with different stopper thickness.

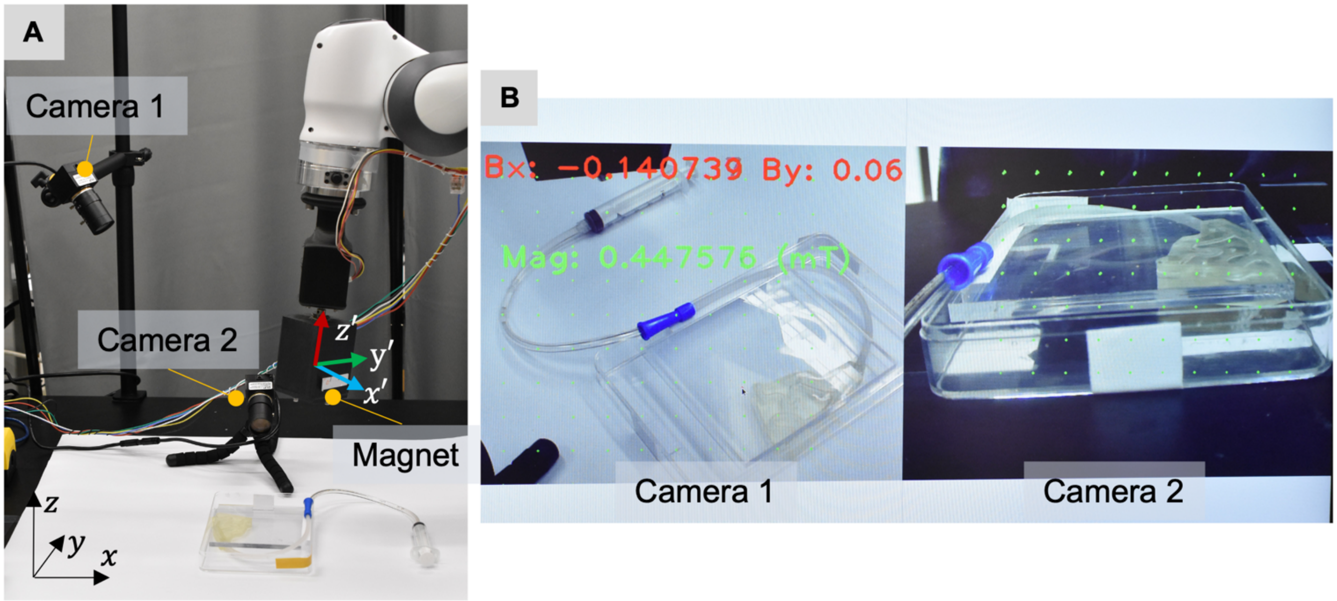

Fig. S11. Robotic arm-based magnetic actuation system. A. Experimental setup for the magnetic actuation. B. Prediction and visualization of the magnetic field in the workspace. Magnetic field unit is in mT and "Mag" indicates the magnitude of the 3D magnetic field.

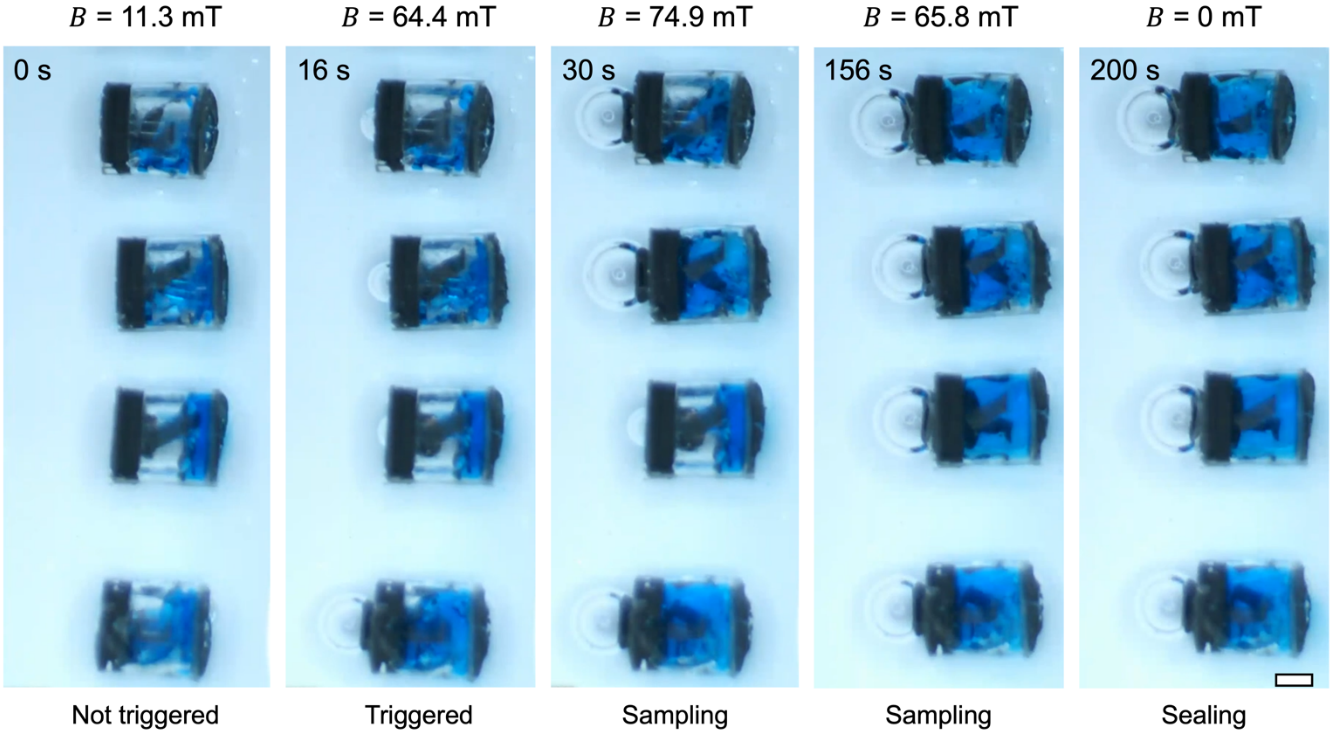

Fig. S12 Demonstration of sampling liquids by a group of soft capsules in an open space with fixed poses. Scale bar, 500 μm.

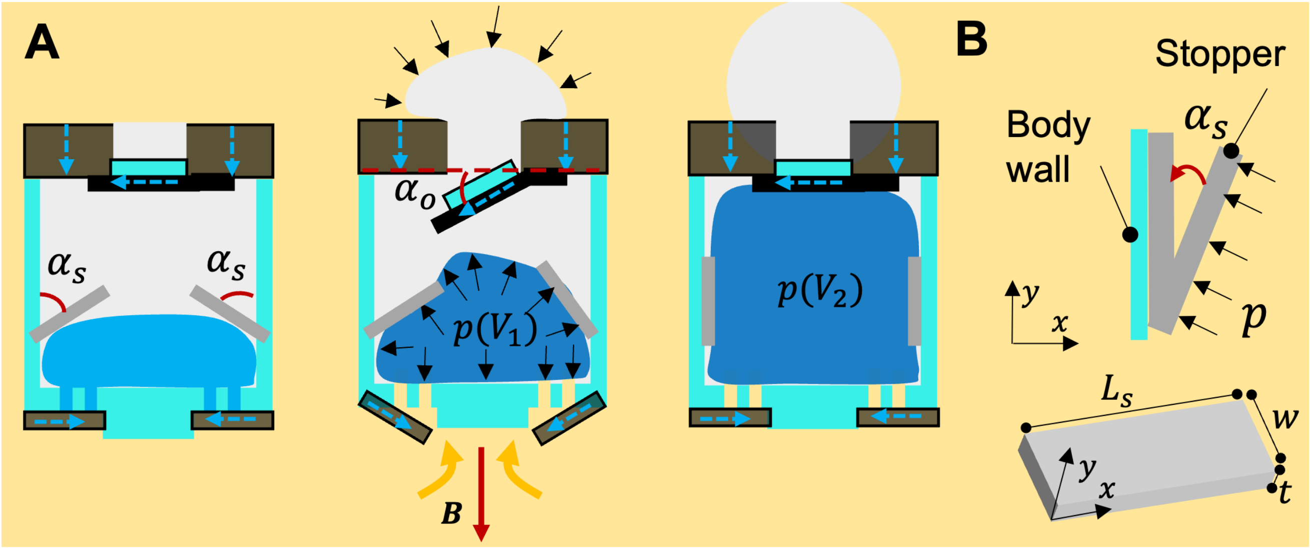

Fig. S13. Illustration of the liquid sampling process. A. Illustration of the swelling process. B. Illustration of the deformation of the stopper by the applied pressure.

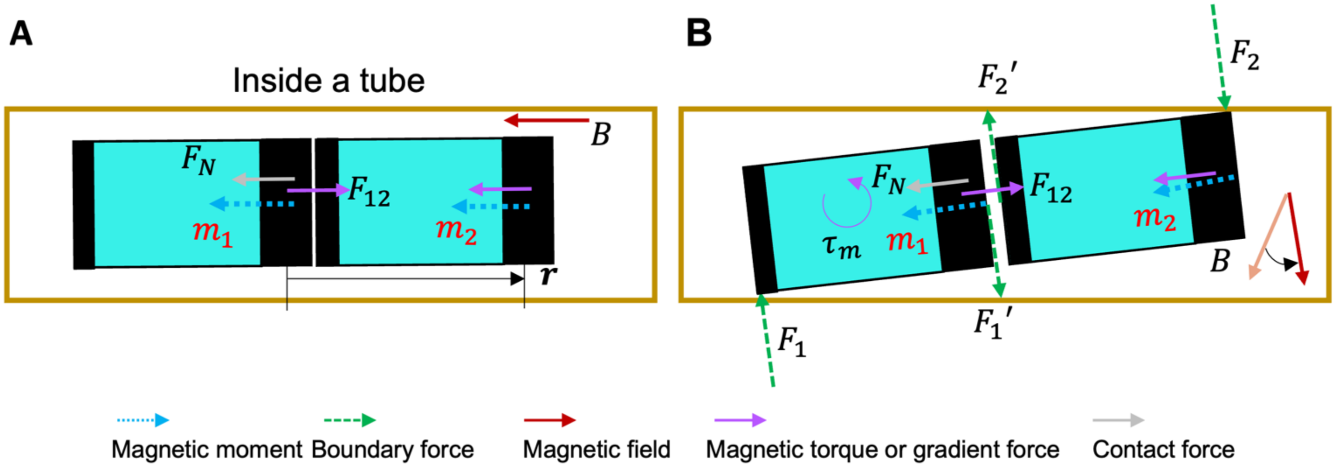

Fig. S14. Analysis of the breaking force for the magnetic capsule chain. A. Schematics of the capsule chain forces when the chain does not touch the boundary wall. B. Schematics of the capsule chain forces when the chain touches the boundary wall.

Supplementary Videos.

Movie S1. Liquid sampling by a soft capsule. This video first shows the swelling of super absorbent polymer without valve control. It then shows the triggering, pumping, and sealing of a millimeter-scale soft capsule inside DI water.

Movie S2. Soft capsule locomotion and delivery, sampling, and retrieval of the soft capsules. This video shows the motion of the capsule in fluid-filled confined spaces, sampling liquids, and capsule retrieval. It also shows the group rolling locomotion and group sampling in open workspace and a confined tubular structure.

Movie S3. Medical imaging guided locomotion and liquid sampling. This video first shows ultrasound imaging-guided locomotion of the soft capsules inside a porcine liver. It then shows the sampling process monitored inside an X-ray cabinet.
